# Characterizing microbial communities and their correlation with genetic mutations in early-stage lung adenocarcinoma: implications for disease progression and therapeutic targets

**DOI:** 10.3389/fonc.2024.1498524

**Published:** 2025-01-07

**Authors:** Hao-Shuai Yang, Jin Zhang, Hong-Xiang Feng, Fei Qi, Fan-Jia Kong, Wei-Jie Zhu, Chao-Yang Liang, Zhen-Rong Zhang

**Affiliations:** Department of Thoracic Surgery, China-Japan Friendship Hospital, Beijing, China

**Keywords:** lung adenocarcinoma, tumor microbiome, PTPRZ1, next-generation sequencing, therapeutic targets

## Abstract

**Background:**

Lung adenocarcinoma (LUAD), the most prevalent form of lung cancer. The transition from adenocarcinoma *in situ* (AIS), and minimally invasive adenocarcinoma (MIA) to invasive adenocarcinoma (IAC) is not fully understood. Intratumoral microbiota may play a role in LUAD progression, but comprehensive stage-wise analysis is lacking.

**Methods:**

Tumor and bronchoalveolar lavage fluid (BALF) samples from patients with AIS/MIA or IAC were collected for next-generation sequencing to characterize microbial diversity and composition. DNA extraction involved lysing samples with nuclease and protease, followed by homogenization and elution. Sequencing libraries were prepared and sequenced on the Illumina platform. Whole exome sequencing was performed to identify somatic mutations and genetic variants. Bioinformatics analysis, including taxonomic annotation with Kraken2 and *de novo* assembly with MEGAHIT, was conducted to process metagenomic data. Correlation analysis was performed to link microbial species with mutated genes using custom R scripts.

**Results:**

Metagenomic analysis revealed a distinct microbial profile in IAC compared to AIS/MIA, with increased abundance of *Bacteroidetes* and *Firmicutes* in the IAC group. *Bosea* sp. and *Microbacterium paludicola*, were less abundant in IAC, suggesting a potential protective role in early-stage disease. Conversely, Mycolicibacterium species were more prevalent in IAC, indicating a possible contribution to disease progression. Genetic sequencing identified PTPRZ1 strongly correlating with microbial composition, suggesting a mechanistic link between microbiota and genetic alterations in LUAD.

**Conclusion:**

This study characterizes microbial communities in various stages of LUAD, revealing links between microbiota and genetic mutations. The unique microbiota suggests its role in LUAD progression and as a therapeutic target.

## 1 Introduction

Lung cancer is the predominant cause of cancer-related deaths globally, with lung adenocarcinoma (LUAD) being the most common subtype (1, 2). It is categorized into stages that reflect its progression, including adenocarcinoma *in situ* (AIS), minimally invasive adenocarcinoma (MIA), and invasive adenocarcinoma (IAC) (3). AIS and MIA represents an early stage, while IAC indicate more advanced stages with increased invasiveness and metastatic potential. Although research has begun to clarify the differences in pathological features (4), imaging signatures (5), and tumor microenvironments (TME) (6) between these stages, the precise mechanisms driving the transition from AIS and MIA to IAC are still not completely understood.

Intratumoral microbiota, the community of microorganisms residing within tumor tissues, is gaining attention for its potential roles in cancer development (7–9). The bacterial load in lung cancer is at an intermediate level among pan-cancers and is enriched with metabolic pathways that degrade chemicals in cigarettes (9). Enteric, potentially pathogenic and pro-inflammatory bacteria were more frequently found in cancer than healthy tissue (10). Multiple studies have revealed differences in microbial composition between lung cancer tissue and non-tumor lung tissue (11). The microbial diversity in non-malignant lung tissue is higher than in tumor tissue, with increased relative abundance of *Thermus* and decreased relative abundance of *Ralstonia* in adenocarcinoma tissue (12). The presence of specific bacteria, such as *Fusobacterium*, has been linked to poor prognosis in lung cancer (13).

Research indicates that the respiratory microbiome potentially influencing the onset and progression of lung cancer through various mechanisms, including inflammatory processes, immune responses, and metabolic regulation (8). The correlation between the composition of intratumoral microbiota and gene mutations was also found in lung cancer (14). Despite the growing body of research, there is a lack of studies focusing on the microbiota in early-stage lung cancer and its progression through different stages of LUAD. Further exploration of the distinct tumor microenvironments at various stages of lung adenocarcinoma is essential for uncovering novel therapeutic targets and improving patient outcomes.

This study focus on the heterogeneity of microbiome within LUAD at various stages to explore the distinct TMEs and uncover new therapeutic targets. To discover candidate bacterial biomarkers and potential relationship between genetic characteristics and progression of lung cancer, we performed sequencing analysis based on tumor and bronchoalveolar lavage fluid (BALF) of patient with AIS/MIA or IAC. Differential microbiota were identified for uncovering novel therapeutic targets and improving patient outcomes.

.

## 2 Materials and methods

### 2.1 Sample collection

A total of 18 patients with lung adenocarcinoma were enrolled from China-Japan Friendship Hospital, and the clinical information is detailed in **Table 1**. Half of them were pathologically diagnosed with AIS or MIA, and other patients were diagnosed with IAC. Formalin-fixed paraffin-embedded (FFPE) samples of tumor tissues and paired adjacent normal tissues from all 18 patients were collected for whole exome sequencing (WES). Tumor tissues and BALF were obtained from 17 patients for metagenomic next-generation sequencing (mNGS). Among these, two tumor tissues and corresponding BALF samples were sequenced for 3 patients with multiple primary nodules. One patient had only BALF sample collected for mNGS sequencing.

**Table 1:** Clinical characteristics of the patients.

Characteristics	All patients (N=18)
Age, years	62.5 (57.0-67.0)
Tumor infiltration	
AIS/MIA	9 (50.0%)
IAC	9 (50.0%)
Sex	
Male	9 (50.0%)
Female	9 (50.0%)
Smoking status	
Never smoker	14 (77.8%)
Former smoker	3 (16.7%)
NA	1 (5.6%)
Basic lung disease	
Yes	2 (11.1%)
No	16 (88.9%)
Nodules	
GGN	17 (94.4%)
Mixed	1 (5.6%)
Previous antibiotic therapy	
Yes	6 (33.3%)
No	12 (66.7%)
Previous hormone therapy	
Yes	1 (5.6%)
No	17 (97.4%)
Disease stage	
TIS	4 (22.2%)
1A1	5 (27.8%)
1A2	9 (50.0%)

Lung cancer patients with different degrees of pathological infiltration underwent surgical resection. During the surgery, tumor tissue, adjacent non-cancerous tissue, and BALF samples were collected. All specimens were stored at −80°C.

### 2.2 DNA extraction

We transferred 2 ml BALF into a centrifuge tube, followed by the addition of lysis reagent and nuclease. The sample was incubated in a constant-temperature metal bath at 37°C for 30 minutes, then at 65°C for 10 minutes. Each fresh tumor tissue sample, approximately the size of a soybean, was placed into a centrifuge tube, and DTT and protease were added. The tube was incubated at 56°C to digest the tissue cells, followed by the addition of lysis reagent and nuclease, with a final incubation at 65°C. Both the alveolar lavage and tissue samples were transferred to lysis tubes. After adding zirconia beads, the tubes were homogenized for 30 minutes utilizing a homogenizer. DNA was eluted with 35 μL of elution buffer, and the quality of extracted DNA was assessed on Qubit^®^ 4.0 fluorometer (Thermo Scientific, Waltham, MA, USA).

### 2.3 Metagenomic next generation sequencing

The construction and sequencing of the DNA library were carried out following the manufacturer’s instructions (Illumina). Extracted DNA was fragmented utilizing a Covaris ultrasonic disruptor to produce fragments of approximately 200 bp. Fragment ends were repaired and A-tailed, followed by ligation with adapters containing barcodes. Subsequently, PCR amplification was performed. The library quality was assessed on Agilent Bioanalyzer 2100 system and quantified on Qubit 4.0 fluorometer (Thermo Scientific, Waltham, MA, USA). The library was sequenced using Nextseq550DX platform in Repugene Technology Co., Ltd (Hangzhou, China).

### 2.4 Whole exome sequencing

Genomic DNA extracted from formalin-fixed paraffin-embedded samples was fragmented into 150-300 bp, with paired tumor and normal tissues. After end-repair of the fragmented DNA and addition of an A-tail, adapters are ligated to both ends of the DNA fragments to construct the DNA library. Libraries with specific indices were pooled and subjected to liquid-phase hybridization using biotin-labeled probes. Exome capture was performed using Agilent SureSelect Human All ExonV6 Kit (Agilent Technologies, Santa Clara, CA, USA). The captured libraries were then linearly amplified by PCR, followed by quality control. DNA sequencing was performed on Illumina NovaSeq 6000 platform (Illumina Inc., San Diego, CA, USA) in Repugene Technology Co., Ltd. (Hangzhou, China), generating 150-bp paired-end reads with mean coverage of 200× for tumor tissue and 100× for adjacent non-cancerous tissue.

### 2.5 Bioinformatics analysis

For the raw data of WES, clean reads were obtained following data filtering and alignment to the human reference genome (GRCh38). Singlenucleotide variants (SNVs) and insertions/deletions were identified using GATK (version 4.4). Additionally, MuTect2 (version 4.1) was employed to detect somatic mutations. To identify structural variants, gene fusion detection was performed using LUMPY (version 0.2.13), and copy number variation (CNV) was detected using CNVkit (version 0.9.9) (15).

For the metagenomic data, Kraken2 (version2.0.7) was employed for unique non-human sequence alignment to annotate microbial species, and count values for all species were normalized as relative abundance. MEGAHIT (version1.2.9) was used for *de novo* assembly of host-filtered sequences, and assembly results were statistically summarized, including final contig sequence lengths and other assembly statistics. MetaGeneMark (version3.38) was utilized for gene prediction on contig sequences from each sample using the MetaGeneMark_v1.mod model. MMseq2 (version2-13.45111) was utilized to cluster homologous genes.

Gene abundances were quantified using Salmon (version1.10.2). For diversity analysis, the vegan package (version 2.6.4) in R was used to compute α-diversity indices, such as Shannon and Simpson indices which reflect the richness and evenness of microbial communities within each sample, and a principal coordinates analysis (PCoA) to provide insights into microbial community differences between groups. Differential abundances of microbiota between groups were analyzed using rank-sum tests. To explore correlations between differential microbiota, Pearson correlations were calculated using the psych package (version 2.3.6). The metacoder package (version 0.3.6) was utilized to visualize the evolutionary relationships of microbial species. Based on the known relationships, taxonomy sets enrichment analysis (TSEA) was employed to explore associations between the microbiota taxa we identified and specific disease. Custom R functions were developed for dimensionality reduction and differential analysis of KEGG Orthology (KO) gene matrices (16). The KEGG KO library was curated, and differential KO genes were analyzed for KEGG enrichment using custom R functions. Spearman correlations between microbiota abundances and KO genes were calculated using the psych package (version2.3.6). Spearman correlations between microbiota abundance and mutation frequencies of genes were assessed using the psych package (version2.3.6).

Mutation-related analysis was conducted using cBioPortal (17) (https://www.cbioportal.org/), a comprehensive open web platform that integrates multiple datasets and offers a range of functions including data mining, data integration, and visualization. For key mutations associated with microorganisms, databases such as CPTAC, OncoSG, and TCGA were jointly applied to explore their mutational characteristics and clinical relevance. The specific analysis steps are displayed in the workflow.

### 2.6 Statistical analysis

Wilcoxon rank sum test was used for microbiota statistical comparison between two groups. The fisher test was performed for mutated genes statistical comparison between two groups. The hypergeometric test was used to assess statistical significance of KO genes enrichment. All statistical analyses were carried out by R (version 4.4.1). Statistical significance was defined as an adjusted P-value < 0.05.

## 3 Results

### 3.1 Tumor mutation profiles and correlation with microbiota

**Figure 1:**
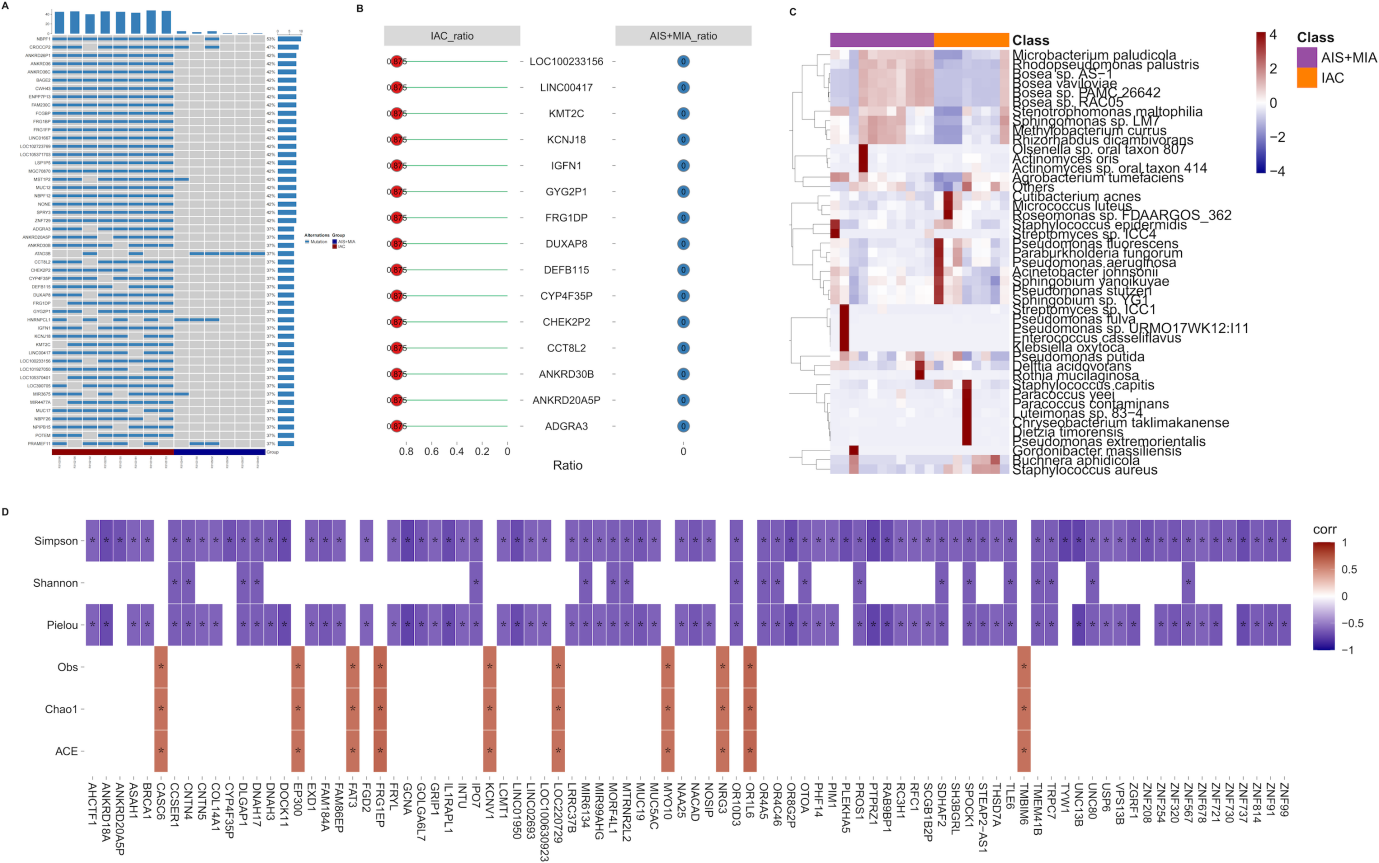
The correlation between mutant landscape and microbiota **(A)** Mutation landscape of the most frequently mutated genes across all samples. **(B)** Top commonly mutated genes in the IAC group. **(C)** Heatmap showing the microbial abundance in AIS/MIA and IAC groups. **(D)** Correlations between α-diversity indices and genes with significantly different mutation frequencies in two clinical groups.(* *p* < 0.05).

Compared to AIS/MIA group, IAC tumors exhibited higher oncogenic mutation frequencies, indicating a heavier tumor mutation burden (**Figure 1A**). For each group, we calculated the proportion of samples with gene mutations out of the total number of samples. Top 15 genes with the highest mutation frequencies in the IAC group was visualized, and these mutations were not detected in AIS/MIA group (**Figure 1B**). The heatmap generated from metagenomic data illustrated different distribution patterns between the groups. Several species, such as *Bosea* sp. *AS-1*, *Bosea* sp. *PAMC 26642*, *Bosea* sp. *RAC05*, *Bosea vaviloviae, Microbacterium paludicola, Rhodopseudomonas palustris*, and *Stenotrophomonas maltophilia*, displayed decreased abundance in IAC group compared with AIS/MIA group (**Figure 1C**). Additionally, genes with differential mutation frequencies between IAC and AIS/MIA groups were selected for downstream analysis. Significant associations between α-diversity indices and mutation frequencies of specific genes suggested a potential link between the microbial community and genetic alternations (**Figure 1D**).

### 3.2 Microbiome variations in IAC and AIS/MIA tumor samples

**Figure 2:**
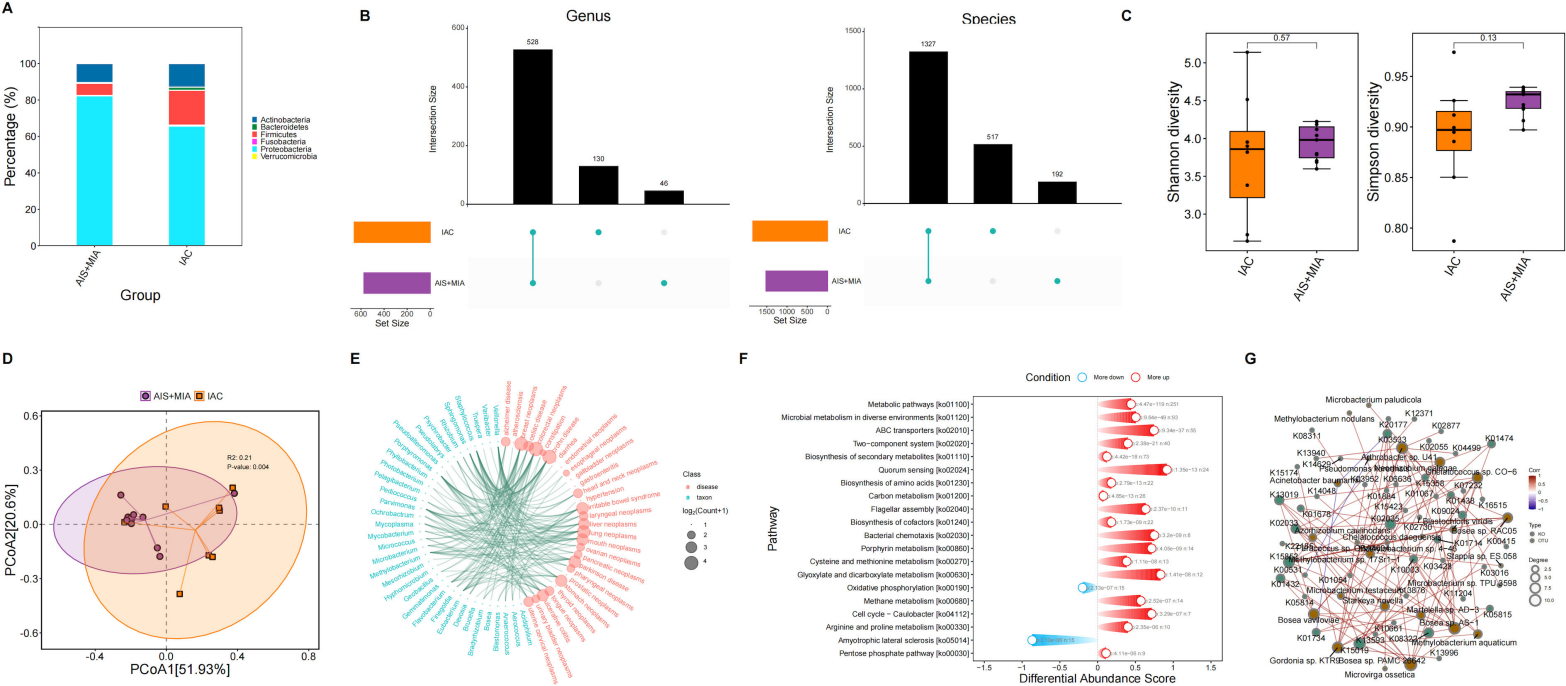
Microbiota characteristics in tumor samples. **(A)** Proportions of microbial phylum in AIS/MIA and IAC groups. **(B)** UpSet diagram showing the microbes detected in AIS/MIA and IAC groups at the genus and species level. **(C)** Scores of α-diversity indices and **(D)** PCoA results in AIS/MIA and IAC groups. **(E)** TSEA visualizing specific microbes associated with diseases, including lung neoplasms. **(F)** Pathways significantly enriched by upregulated or declined KO genes in AIS/MIA group. **(G)** The interaction network showing the correlations between microbes with significantly different abundance and genes with significantly different mutation frequencies in two groups.

We identified six predominant microbial phylum in the tumor microenvironment, including *Actinobacteria, Bacteroidetes, Firmicutes, Proteobacteria, Verrucomicrobia*, and *Fusobacteria*. Increased proportions of *Bacteroidetes* and *Firmicutes* were observed in IAC group rather than AIS/MIA group (**Figure 2A**). At the genus level, 130 and 46 unique genera were identified in IAC and AIS/MIA groups, respectively, with 528 genera shared between the two groups. At the species level, 517 and 192 unique species were identified in IAC and AIS/MIA groups, respectively, and 1327 species were shared by the two groups (**Figure 2B**). Subsequently, α-diversity analysis revealed that IAC group was characterized by higher Shannon diversity and Simpson diversity scores than AIS/MIA group, despite with no significant difference (**Figure 2C**). Additionally, PCoA plot showed significant differences in tumor microbiota between IAC and AIS/MIA groups (P = 0.004) (**Figure 2D**). Several microbial genera detected in this study have previously been reported to be involved in lung cancer development, as revealed TSEA analysis, such as *Blastomonas, Gemmatimonas, Mesorhizobium, Microbacterium, Mycobacterium, Mycoplasma, Parvimonas, Porphyromonas, Sphingomonas, Staphylococcus*, and *Veillonella* (**Figure 2E**). Additionally, KO genes with increased abundance in the AIS/MIA group were significantly enriched in signaling transduction pathways, such as ABC transporters and bacterial chemotaxis, and metabolic pathways (**Figure 2F**). Subsequently, we explored interactions between microbes with significantly different abundance and genes with significantly different mutation frequencies. As a result, the interaction network uncovered the extensively positive correlations between specific microbes and KO genes, including *Bosea* sp. *PAMC 26642* with K01432 and K02035, and *Bosea* sp. *RAC05* with K01438 (**Figure 2G**).

### 3.3 Microbiome variations in IAC and AIS/MIA BALF samples

**Figure 3:**
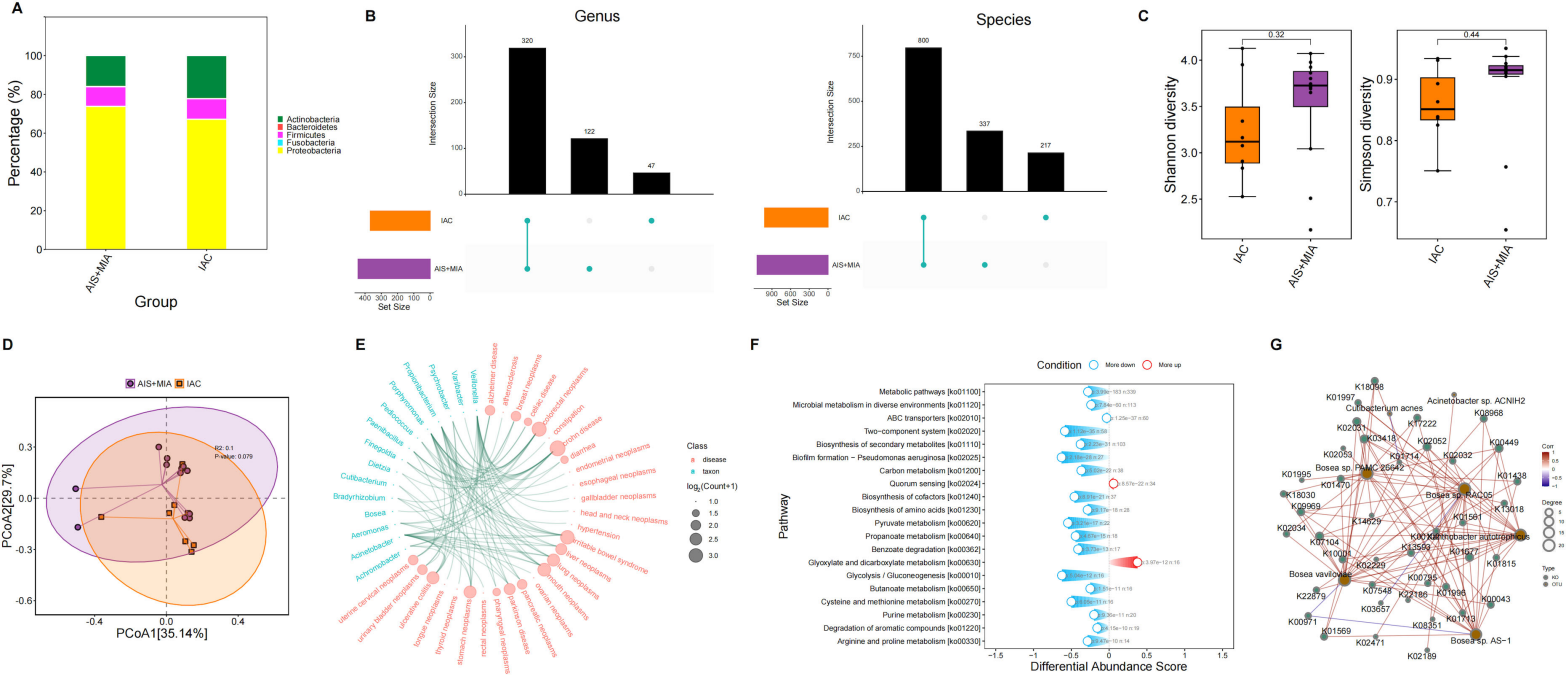
Microbiota characteristics in BALF samples. **(A)** Proportions of microbial phylum in AIS/MIA and IAC groups. **(B)** UpSet diagram showing the microbes detected in AIS/MIA and IAC groups at the genus and species level. **(C)** Scores of α-diversity indices and **(D)** PCoA results in AIS/MIA and IAC groups. **(E)** TSEA visualizing specific microbes associated with diseases, including lung neoplasms. **(F)** Pathways significantly enriched by upregulated or declined KO genes in AIS/MIA group. **(G)** The interaction network showing the correlations between microbes with significantly different abundance and genes with significantly different mutation frequencies in two groups.

Five predominant microbial phylum in the BALF microenvironment were detected, including *Actinobacteria, Bacteroidetes, Firmicutes, Proteobacteria*, and *Fusobacteria*. Compared to AIS/MIA group, IAC group showed increased *Actinobacteria* but declined *Proteobacteria* (**Figure 3A**). At the genus level, 47 and 122 unique genera were identified in IAC and AIS/MIA groups, respectively, with 320 genera shared between the two groups. At the species level, 217 and 337 unique species were identified in IAC and AIS/MIA groups, respectively, and 800 species were shared by the two groups (**Figure 3B**). Although there was no significant difference, we obtained higher Shannon diversity and Simpson diversity scores in AIS/MIA group rather than IAC group (**Figure 3C**). Slight difference between IAC and AIS/MIA groups was revealed by PCoA plot, and statistically significance did not reached (**Figure 3D**).

Based on the known relationships between microbes and specific diseases, we performed TSEA analysis, which suggested the involvement of some genus in lung cancer development, such as *Achromobacter, Acinetobacter, Porphyromonas, Propionibacterium*, and *Veillonella* (**Figure 3E**). KO genes with increased abundance in the IAC group were significantly enriched in more pathways associated with metabolism and signaling transduction, such as carbon metabolism, two-component system, and biosynthesis of cofactors (**Figure 3F**). Subsequently, we explored the interactions between microbes with significantly different abundance and genes with significantly different mutation frequencies. The interaction network uncovered the extensively positive correlations between specific microbes and KO genes, such as *Bosea* sp. *PAMC 26642* with K03418 and K00449, and *Bosea* sp. *RAC05* with K02031 (**Figure 3G**).

### 3.4 Key microbial identification and functional speculation

**Figure 4:**
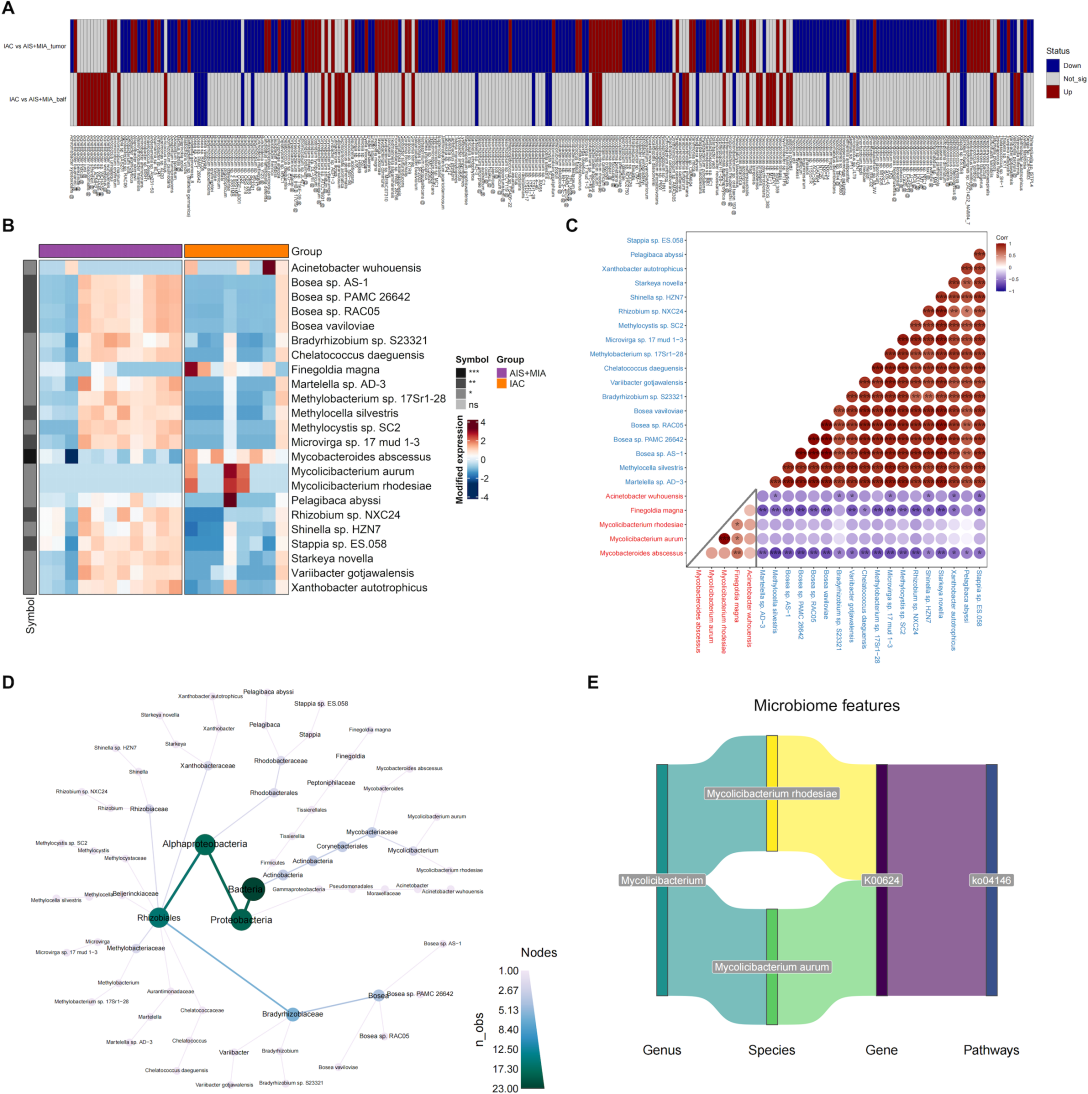
Identification of key microorganisms in tumors and BALF **(A)** Microbial species with significantly altered relative abundance in the IAC group compared to the AIS group in tumor or BALF samples. **(B)** Heatmap showing the abundance of microbes that are significantly upregulated or downregulated in both tumor and BALF samples of IAC group. The correlation matrix **(C)** and evolutionary tree **(D)** of the 23 microbes that exhibited consistent upregulation or downregulation in IAC group across both sample types. **(E)** The potentially regulatory axis of Mycolicibacterium. (* *p* < 0.05; ** *p* < 0.01; *** *p* < 0.001).

For the microbes with significantly different abundances between IAC and AIS/MIA groups, we visualized their profiles in two types of samples: tumor and BALF (**Figure 4A**). A total of 23 microbes exhibited consistent upregulation or downregulation in IAC group across both sample types (**Supplementary Table S1**). Specifically, five microbes increased in IAC group, including *Mycobacteroides abscessus*, *Mycolicibacterium aurum*, *Mycolicibacterium rhodesiae*, *Finegoldia magna*, and *Acinetobacter wuhouensis*, while the remaining 18 microbes exhibited decreased abundance in IAC group (**Figure 4B**). The correlations among these 23 microbes were subsequently investigated. As expected, negative correlations were observed between upregulated and downregulated microbes, indicating potential antagonistic relationships. Of note, *Mycobacteroides abscessus*, which was upregulated in IAC group, displayed negative associations with all 18 declined microbes, suggesting its powerful influence (**Figure 4C**). The phylogenetic tree generated from the taxonomy analysis indicated that these 23 microbes primarily belonged to *Proteobacteria*, *Alphaproteobacteria*, and *Rhizobiales taxa* (**Figure 4D**). Among the five upregulated microbes in IAC group, two of them belonged to *Mycolicibacterium* genus, including *Mycolicibacterium rhodesiae* and *Mycolicibacterium aurum*. To further characterize their roles, we performed correlation analysis among microbes, KO genes, and KO pathways, and revealed the axes among *Mycolicibacterium rhodesiae/Mycolicibacterium aurum* KO0624 gene-KO04146 pathway, suggesting the potentially regulatory roles (**Figure 4E**).

### 3.5 PTPRZ1 is a key mutation associated with microorganisms

**Figure 5:**
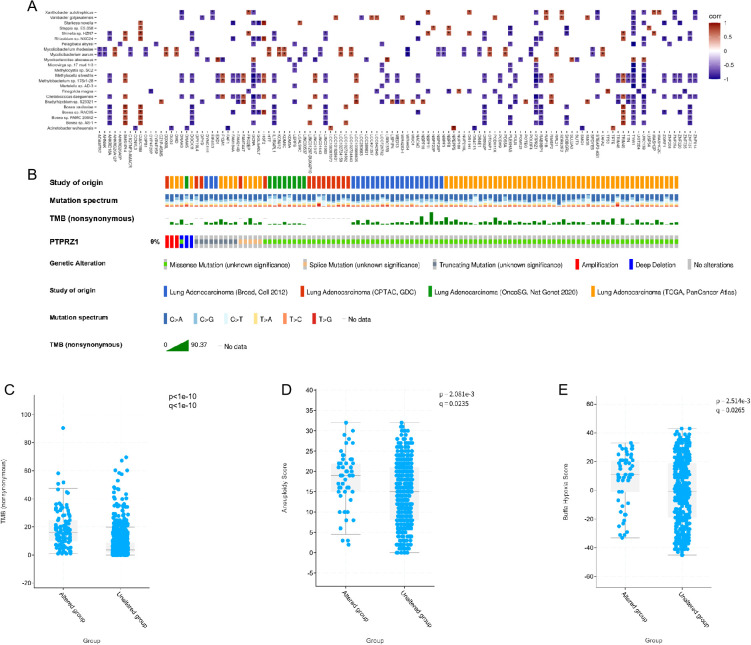
Microbial related mutations and clinical correlation analysis. **(A)** Correlations between the 23 microbes and genes with significantly different mutation frequencies in IAC compared to AIS/MIA group. **(B)** Mutation landscape of PTPRZ1 in multiple lung adenocarcinoma databases. **(C)** Correlation analysis between PTPRZ1 and tumor mutation burden. **(D)** Correlation analysis between PTPRZ1 and aneuploidy score. **(E)** Correlation analysis between PTPRZ1 and buffa hypoxia score. (* *p* < 0.05; ** *p* < 0.01; *** *p* < 0.001).

To investigate the dysregulated characteristics within tumor microenvironment, we performed a correlation analysis between mutated genes and 23 significant microbes. Significant correlation values were widely observed between key microbes and mutated genes (**Figure 5A**). Of note, the strong correlations of TYW1, PTPRZ1, and GCNA genes with more than 20 different microorganisms suggested that these genes may play an crucial regulatory role in the microbial community of the lung. Among them, previous studies have found that PTPRZ1 is closely related to the occurrence and development of lung cancer. Through the joint analysis of multiple databases, the mutation situation of PTPRZ1 in lung adenocarcinoma is shown in **Figure 5B**. Mutations in the PTPRZ1 protein are closely related to TMB, Aneuploidy score, and Buffa hypoxia score (**Figures 5C-E**).

## 4 Discussion

The intricate relationship between the lung microbiome and LUAD has been a subject of increasing interest in cancer research (7, 18, 19). Our study builds upon previous work by examining the heterogeneity of microbiota within LUAD at various stages, focusing on the transition from AIS/MIA to IAC. This progression is characterized by a shift in the microbiota composition, which may reflect alterations in the tumor microenvironment (TME) that facilitate tumor growth.

Research has consistently highlighted that the development of lung cancer is a complex process, influenced by a multitude of factors including tobacco smoking, immune responses, viral infections, and more (20). Prior studies have indicated that the microbiome plays a significant role in the intricate dance of lung cancer progression (21), contributing in various specific manners to this multifaceted disease. Our findings are in line with prior research that has identified specific microbial signatures associated with LUAD. For instance, studies have reported an increase in the abundance of *Proteobacteria* (22) and *Firmicutes* (23) in lung cancer tissues compared to non-cancerous lung tissues. Similarly, our results show an increase in *Bacteroidetes* (24) and *Firmicutes* in IAC, suggesting these phyla may be associated with a more aggressive tumor phenotype. Research has found that *Firmicutes* may promote the proliferation and angiogenesis of lung cancer cells through the action of Th17 cells (25). The decrease in certain bacterial species such as *Bosea* sp. and *Microbacterium paludicola* in IAC, as observed in our study, aligns with research indicating these microbes may have tumor-suppressive properties or are outcompeted in a more aggressive TME.

Our study also highlights the potential of microbiota as a therapeutic target in LUAD. The differential abundance of microbes in IAC versus AIS/MIA suggests that certain microbial species may promote or inhibit tumor progression. Through joint analysis of tumor tissue and BALF, we have identified that *Mycolicibacteria* species are key differential strains between IAC and MIA/AIS, and may play an important role in the evolution of lung cancer. The increased abundance of *Mycolicibacterium* (26)species in IAC could indicate a role in creating a pro-tumorigenic environment, as previously suggested for other cancers, such as pancreatic cancer and melanoma (9, 27). The functional speculation based on key microbial identification points towards a complex interplay between the microbiota and LUAD pathogenesis. The upregulation of *Mycolicibacterium* species in IAC may indicate a role in promoting a pro-tumorigenic environment, where these microbes have been associated with inflammation and immune evasion, chronic infections associated with *Mycobacteria* may increase the risk of lung cancer (28). The negative correlations observed between *Mycobacteroides abscessus* and other microbes suggest a potential competitive interaction within the TME, which could be pivotal in LUAD progression. Targeting these microbes or their metabolic pathways could offer a novel therapeutic strategy.

The microbiome, through its metabolic activities, can produce compounds that may affect gene expression and contribute to genomic instability, thus potentially impacting cancer development (29). The correlation between PTPRZ1 mutations and microbial species is a novel finding that warrants further exploration. PTPRZ1, also known as receptor-type tyrosine-protein phosphatase, is a transmembrane protein that plays a crucial role in cell adhesion, migration, and signal transduction (30, 31). It has been implicated in various cellular processes, including cell growth regulation and the maintenance of tissue integrity in glioblastoma (32). In the context of LUAD, PTPRZ1 has been suggested to be involved in the modulation of cell adhesion and migration, which are critical steps in tumor progression and metastasis (33). Our recent data indicate that mutations in PTPRZ1 may be correlated with the presence of specific microbial communities within the lung environment. This correlation is particularly intriguing as it suggests a potential interaction between genetic mutations and the microbiome, which could influence the development and progression of LUAD. The presence of certain microbial species, such as *Mycobacteroides* may create a microenvironment that either promotes or suppresses the effects of PTPRZ1 mutations. For instance, microbial metabolites could interact with PTPRZ1, altering its function and thereby affecting cellular processes such as cell adhesion and migration (34). This interaction could lead to a more aggressive phenotype in LUAD, characterized by increased invasiveness and metastasis.

It is important to acknowledge the limitations of our study. The cross-sectional design limits our ability to infer causality and the temporal dynamics of microbial changes during LUAD progression. Additionally, the microbial taxa identified require further validation in larger and longitudinal samples to track their changes over the cancer progression to confirm their role in LUAD development. The use of BALF and tumor tissue samples provides a snapshot of the microbiota but may not capture the full complexity of microbial communities in the lung. This study is a single center study with a small sample size, potentially limiting the ability to detect effects and possibly affecting the statistical power of the results. Caution is exercised in interpreting the findings, with an emphasis on the need for more data to support the conclusions. The results are considered preliminary and require further validation through larger-scale studies.

## 5 Conclusion

This study reveals distinct microbial profiles associated with the progression of lung adenocarcinoma, with potential implications for disease prognosis and therapy. The findings suggest that specific microbial species may promote or inhibit tumor progression, and a correlation between genetic mutations, such as in PTPRZ1, and microbial composition offers a novel perspective on LUAD pathogenesis. These insights could lead to new diagnostic and therapeutic strategies targeting the tumor microbiome.

## Data Availability

The original contributions presented in the study are included in the article/**Supplementary Material**. Further inquiries can be directed to the corresponding authors.
