# Supplementary figures and images for: Characterizing microbial communities and their correlation with genetic mutations in early-stage lung adenocarcinoma: implications for disease progression and therapeutic targets
Source: Front Oncol. 2025 Jan 7;14:1498524. doi: 10.3389/fonc.2024.1498524 (PMC11752883; doi:10.3389/fonc.2024.1498524)

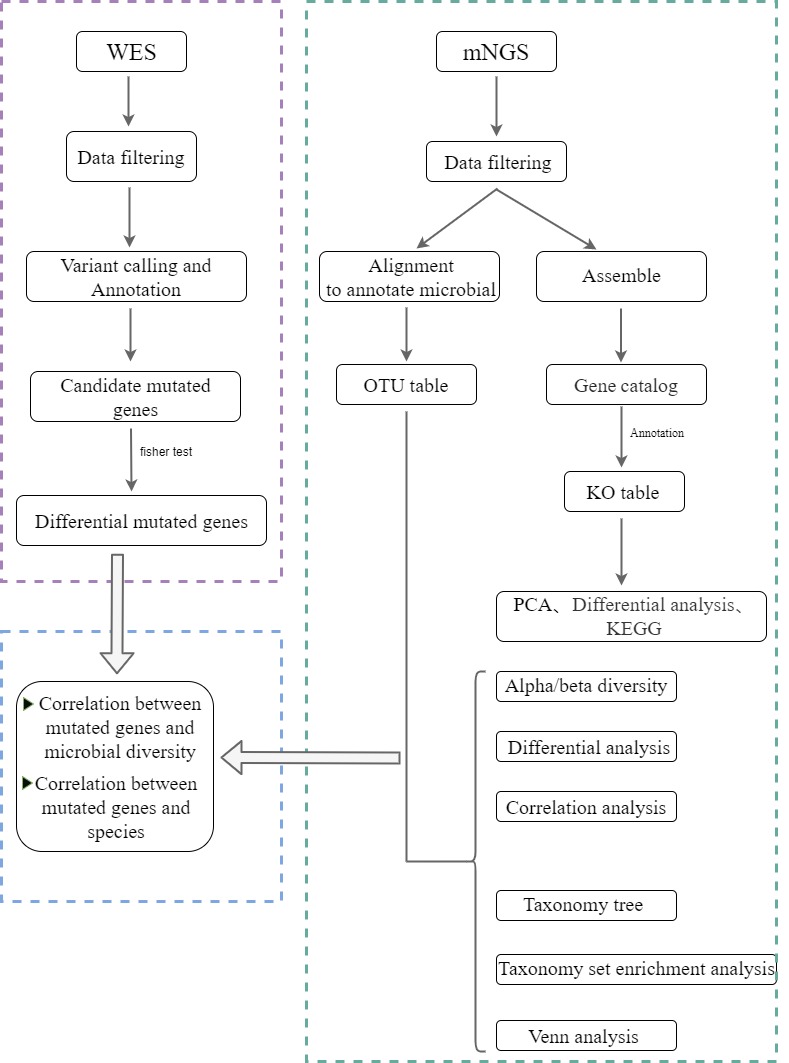

Supplement: Supplementary file 1 [file Image1.jpeg]
