# Supplementary material for: Characterizing microbial communities and their correlation with genetic mutations in early-stage lung adenocarcinoma: implications for disease progression and therapeutic targets
Source: Front Oncol. 2025 Jan 7;14:1498524. doi: 10.3389/fonc.2024.1498524 (PMC11752883; doi:10.3389/fonc.2024.1498524)
Supplement: Supplementary file 2 [file Table1.docx]

| IAC_Up | IAC_Down | | |
| --- | --- | --- | --- |
| Mycobacteroides abscessus | Martelella sp. AD-3 | Bradyrhizobium sp. S23321 | Rhizobium sp. NXC24 |
| Mycolicibacterium aurum | Methylocella silvestris | Variibacter gotjawalensis | Shinella sp. HZN7 |
| Mycolicibacterium rhodesiae | Bosea sp. AS-1 | Chelatococcus daeguensis | Starkeya novella |
| Finegoldia magna | Bosea sp. PAMC 26642 | Methylobacterium sp. 17Sr1-28 | Xanthobacter autotrophicus |
| Acinetobacter wuhouensis | Bosea sp. RAC05 | Microvirga sp. 17 mud 1-3 | Pelagibaca abyssi |
|  | Bosea vaviloviae | Methylocystis sp. SC2 | Stappia sp. ES.058 |

Table S1 23 microbes exhibited consistent upregulation or downregulation in IAC group across both sample types
